# Estimating whole-body centre of mass sway during quiet standing with inertial measurement units

**DOI:** 10.1371/journal.pone.0315851

**Published:** 2025-01-13

**Authors:** Liam H. Foulger, Emma R. Reiter, Calvin Kuo, Mark G. Carpenter, Jean-Sébastien Blouin

**Affiliations:** 1 School of Kinesiology, University of British Columbia, Vancouver, British Columbia, Canada; 2 School of Biomedical Engineering, University of British Columbia, Vancouver, British Columbia, Canada; 3 Centre for Hip Health and Mobility, University of British Columbia, Vancouver, British Columbia, Canada; 4 Djavad Mowafaghian Centre for Brain Health, University of British Columbia, Vancouver, British Columbia, Canada; 5 Institute for Computing, Information and Cognitive Systems, University of British Columbia, Vancouver, British Columbia, Canada; Universita Politecnica delle Marche Facolta di Ingegneria, ITALY

## Abstract

Our ability to balance upright provides a stable platform to perform daily activities. Balance deficits associated with various clinical conditions may affect activities of daily living, highlighting the importance of quantifying standing balance in ecological environments. Although typically performed in laboratory settings, the growing availability of low-cost inertial measurement units (IMUs) allows the assessment of balance in the real world. However, it is unclear how many IMUs are required to adequately estimate linear displacements of the centre of mass (CoM) at stance widths associated with daily activities. While wearing IMUs on their head, sternum, back, right thigh, right shank, and left shank, 16 participants stood quietly on a force platform in narrow, hip-width, and shoulder-width stances, each for three two-minute trials. Using a multi-segment biomechanical model, we estimated CoM displacements from all possible combinations of the IMUs. We then calculated the correlation between the IMU- and force platform- CoM estimates to determine the minimal number of IMUs needed to estimate CoM sway. Four IMUs were necessary to accurately estimate anteroposterior (AP) and mediolateral (ML) CoM displacements across stance widths. Using IMUs on the back, right thigh, and both shanks, we found strong correlations between the IMU CoM estimation and the force platform CoM estimation in narrow stance (AP: r = 0.92±0.04, RMSE = 2.39±2.08 mm; ML: r = 0.97±0.02, RMSE = 1.16±0.77 mm), hip-width stance (AP: r = 0.93±0.04, RMSE = 2.00±1.18 mm; ML: r = 0.92±0.06, RMSE = 0.92±0.70 mm), and shoulder-width stance (AP: r = 0.93±0.03, RMSE = 1.95±1.66 mm; ML: r = 0.86±0.13, RMSE = 1.39±1.46 mm). These results indicate that IMUs can be used to estimate CoM displacements during quiet standing and that four IMUs are necessary to do so. Using an algorithm based on a simple biomechanical model, researchers and clinicians can estimate whole-body CoM displacements accurately during unperturbed quiet standing. This approach can improve the ecological validity of standing balance research and opens the possibility for assessing/monitoring patients with standing balance deficits.

## Introduction

Our ability to balance upright provides a stable platform to perform many activities of our day-to-day life. Traditionally, standing balance assessments have been performed in research laboratories using specialized equipment [1]; however, the growing availability of low-cost inertial measurement units (IMUs) allows us to quantify balance in the real world [2]. While many authors have correlated summary statistics of body sway using IMUs to gold standard measures [2–9], these often involve comparisons between different metrics, e.g., accelerations measured from IMUs and displacements measured from force platforms or motion capture.

Whole-body sway when balancing upright is typically characterized by measuring the centre of mass displacement (CoM) [10]. Theoretically, linear acceleration signals from IMUs could be integrated twice to obtain linear displacements, but in practice, the acceleration estimates are noisy and their biases result in displacement drifts over time [11]. Alternatively, we can fuse accelerometer and gyroscope information to estimate the orientation (i.e., pitch and roll) of the IMU accurately [12]. In anteroposterior (AP) body sway, the body generally acts as a single rigid-link inverted pendulum with all body segments above the ankle swaying together, so the body angle can be converted to a CoM displacement [13, 14]. The biomechanics of mediolateral (ML) sway behaviour; however, involve at least a quadrilateral model for the lower limbs and an additional trunk segment to account for possible counter-rotation between the upper and lower body segments depending on stance width [15–18]. In this case, IMUs may be required on multiple segments to adequately capture the biomechanics of ML balance. Some authors have successfully estimated postural oscillations for a single stance width using IMUs, but their approaches involved a fixed number of IMUs or were validated only for short durations (≤ 30 seconds) [19, 20]. Given that clinical balance assessments can occur at different stance widths [1], it is critical to determine both the minimal number of IMUs required to estimate body sway and how this number may change with stance width.

The objective of this study was to determine the minimum number of IMUs needed to adequately estimate CoM sway during quiet standing across common stance widths and to provide open-source software for researchers/clinicians to do so. We developed an algorithm based on existing biomechanical models and anthropometric data to estimate CoM sway with up to six IMUs placed across the body. We estimated CoM sway with various combinations of IMUs and correlated the results with the CoM sway estimated from a force platform. Due to the common kinematics of most segments for AP balance, we expected that only one IMU on the lower limb or back would be required to estimate CoM sway in that direction across stance widths. We predicted that IMUs from the lower and upper body would be required to estimate CoM in the ML direction due to the potentially distinct kinematics of these segments across stance widths.

## Methods

### Participants

Sixteen adult participants [8 females, 8 males; age 25±3 years, mass 74±15kg, height 174±12cm (mean ± SD)] with no neurological or musculoskeletal impairments were recruited between September 20, 2022 and October 30, 2022, and provided written informed consent. The experiments were reviewed by the University of British Columbia’s Clinical Research Ethics Board (H22-01772).

### Set-up

**Fig 1:**
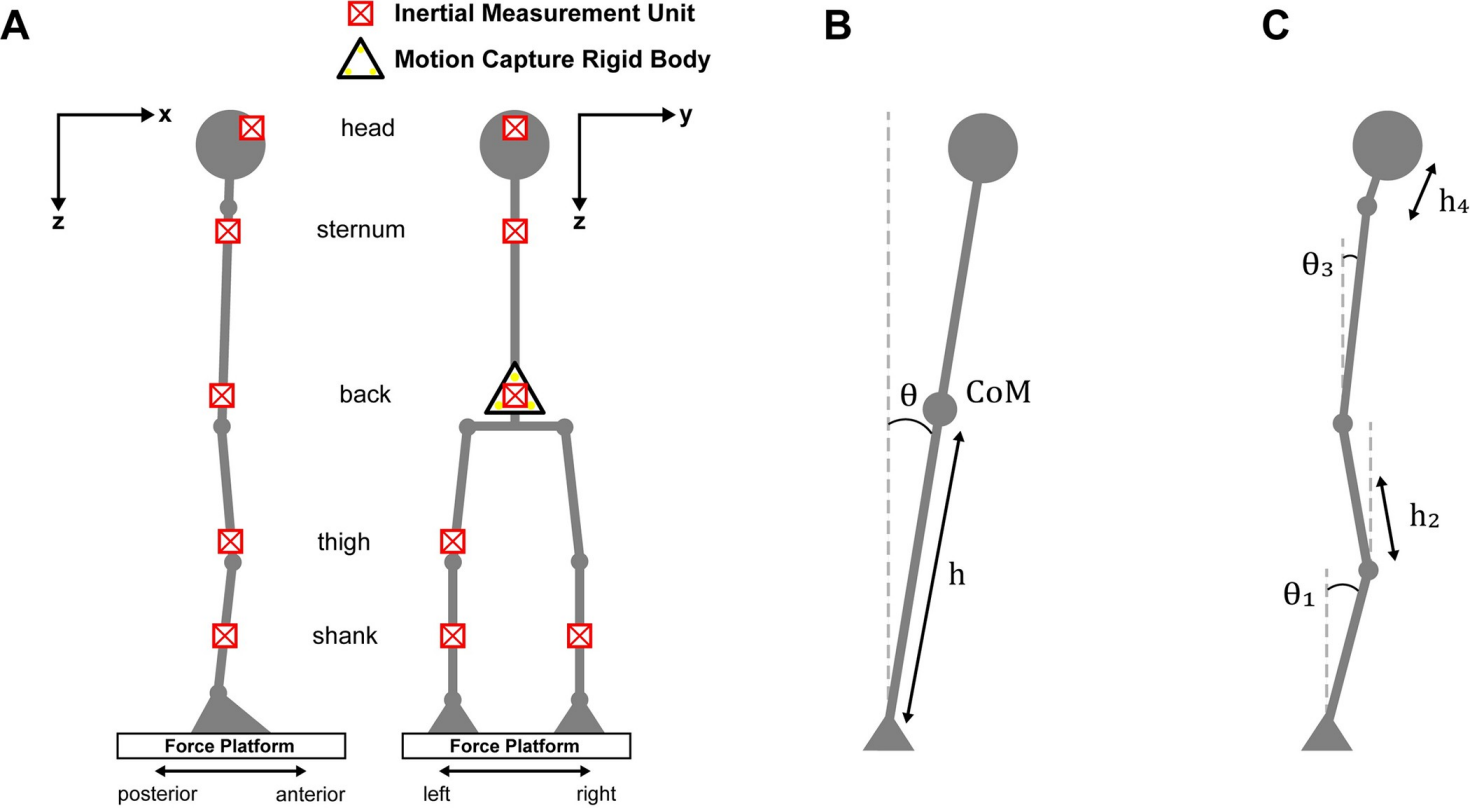
Experimental set-up and balance models. (A) Sagittal (left) and posterior (right) views of the inertial measurement unit (IMU) placements and coordinate reference frame for data collection. (B) Single-segment inverted pendulum balance model. CoM: centre of mass; h: centre of mass height; θ: total pendulum orientation. (C) Multi-segment inverted pendulum balance model. When segment orientations were excluded from the multi-segment model (due to the omission of the IMU data), the segment sway was determined by the sway of the closest segment with an IMU. We split the segments depending on their location with respect to the hip joint when necessary (e.g., if IMUs on the shank and back were used, the thigh segment sway was determined by the shank sway, given they are both below the hip joint) or chose the most inferior segment (e.g., if the head and back IMUs were used, the sternum segment sway was determined by the back sway). *h*_*i*_: segment centre of mass height; *θ*_*i*_: segment orientation.

We attached six IMUs (MPU 6050; range: ±2g and ±250˚/s) to the participant, positioned on their: 1) forehead (~5cm above the glabella); 2) manubrium (below the suprasternal notch); 3) back (over the L3 vertebra to represent the approximate location of whole body CoM [21, 22]); 4) right lateral thigh (7±2cm SD above the lateral tibiofemoral joint line); and 5–6) anterior crest of both tibias (halfway between the medial malleolus and the tibial tuberosity). Only the right thigh was instrumented with an IMU because our set-up was limited to six IMUs and we predicted that the movement of the thighs would not largely differ from the shanks given that the knees are considered to be locked when modelling standing balance [23]. We calibrated all IMUs and standardized their local coordinate reference frames to a body reference frame of X: forward, Y: right, and Z: down [24] (Fig 1A; see “Sensor Calibration”).

We placed the back IMU on a 7cm equilateral triangle rigid body with three optical tracking markers (Optotrak Certus 3020, Northern Digital Inc, Canada) which was positioned on the lower back. The optical markers were used to provide an estimate of body CoM linear displacement to compare with the force plate and IMU estimates. This location was chosen because lumbar and whole-body CoM linear displacements are highly correlated during quiet standing [13]. The optical markers were also used to validate the IMU orientation algorithm (see S1 File).

### Sensor calibration

We calibrated all IMUs to standardize their local coordinate reference frames to a standard body reference frame of X: forward, Y: right, and Z: down. The calibration was achieved by recording five seconds of two static poses for each sensor after it was placed on the body. For the first pose, the participant was in an upright position to define the Z-axis as the gravitational vector from the accelerometer. For the second pose, participants pitched each respective body segment ~90 degrees forward and down to define an approximate X-axis facing forward. To determine the approximate X-axis and true Z-axis, the axes were defined as opposite to the net acceleration vector caused by the gravitational field. The Y-axis was defined as the cross product of the Z-axis and the approximate X-axis, and the true X-axis was determined by the cross product of the Y- and Z-axes [24].

### Protocol

Participants stood on a force platform (OR6-7, AMTI, USA) and were instructed to stand normally with their hands at their sides and to look at a target ~1 metre away placed at eye level. The force platform and the six degrees-of-freedom IMU data were acquired at 250 Hz with a real-time embedded controller (CompactRIO-9082 with NI-9205 analog input and NI-9402 digital input/output modules; NI, USA) running a custom-made LabVIEW (LabVIEW 2019b; NI, USA) virtual instrument. Optical motion tracking data were also collected at 250 Hz with a custom-made MATLAB program (2007, The MathWorks Inc, USA).

We tested three stance conditions (50%, 100%, and 150% of hip joint width), corresponding to narrow (feet almost touching), hip-, and shoulder-width foot stances, respectively. Participant hip joint width was estimated using pelvic width and pelvic depth measurements [25]. We measured the distance between the middle of the tali for each stance condition and marked feet positions to ensure repeatability between trials. The order of stance conditions was randomized, and each was completed in blocks of three 120-second trials.

### Data processing

We performed data processing and analysis using custom scripts written in MATLAB 2023a. All data were time-synchronized, interpolated, and low-pass filtered at 10 Hz (Butterworth, dual-pass fourth-order). We estimated whole-body CoM position from the force platform data (*CoM*_*FP*_) using the zero-point-to-zero-point double integration technique [26, 27], low-pass filtered at 10 Hz (Butterworth, dual-pass fourth-order) to remove discontinuities caused by the double integration technique. The zero-point-to-zero-point double integration technique allows whole-body estimation of CoM with only a force platform, and previous work has shown root mean square errors between this approach and a 13-segment motion capture approach to be < 1 mm in both AP and ML [27].

### Multi-segment CoM model

We used a multi-segment biomechanical model to estimate the CoM of the individual body segments corresponding to the placement of the IMU(s) (Fig 1B, 1C). We calculated whole-body CoM sway by summing individual segment sway, weighted by their relative mass. This approach allows for IMU placement flexibility: if a given body segment does not have an IMU, segment sway is determined by the closest segment with an IMU (Fig 1C). Another advantage of this model is that it only requires the participant’s total height to be measured; individual segment lengths and mass ratios were determined using existing anthropometric data based on participant sex and height [22].

### IMU orientation & CoM estimation

We estimated the CoM displacement of each segment using the accelerometer and gyroscope from an IMU. First, we determined the orientation of the IMU using a complementary filter [12] (see S1 File). After determining the orientation of each segment in the pitch and roll axes, we estimated the displacement of the segment CoM (***CoM***_***i***_) with Eq (1), where ***h***_***i***_ is the relative CoM height of the segment, ***θ***_***i***_ is the segment orientation, ***l***_***n***_ and ***θ***_***n***_ are respectively the total length(s) and orientation(s) for the segment(s) below (when applicable). Finally, we calculated whole-body CoM (***CoM***_***IMU***_) with Eq (2) given the relative segment masses (***m***_***i***_).


$$CoM_{i}=h_{i}sin\theta_{i}+\sum_{n=1}^{i-1}l_{n}\;\sin\;\theta_{n}$$ (1)

$${CoM}_{IMU}=\sum m_{i}CoM_{i}$$ (2)

### Statistical analysis

To determine the minimal number of IMUs needed to estimate CoM, we compared the correlations between the *CoM*_*IMU*_ and *CoM*_*FP*_ to the correlations between the back linear displacement from the motion capture (*CoM*_*mocap*_) and the *CoM*_*FP*_, because trunk linear displacement is strongly correlated with whole-body CoM sway [13]. For each stance width, we identified the best IMU combination, ranging from one to six IMUs, based on the highest average Pearson’s correlation coefficient between the AP and ML directions. If either the AP or ML correlation coefficients from a desired number of IMUs were < 0.8, this combination of IMUs was excluded from the analysis. The combinations of AP and ML IMUs did not have to match as long as the total number of unique IMUs was equal to or below the evaluated number of IMUs. Then, we compared the AP and ML correlations from the chosen combinations from each number of IMUs to those obtained from the *CoM*_*mocap*_−*CoM*_*FP*_ correlations using one-sided paired samples student t-tests (p<0.05). Prior to the statistical tests, we applied a Fisher transformation for all analyses to correct for the non-normality of correlation coefficients. When no significant difference between the motion capture and IMU correlations was found, we determined this IMU combination to be the minimal number of IMU needed to estimate the CoM in that stance width and direction. We also performed comparisons to determine if adding additional IMUs could improve the CoM estimates, or to determine which combination of IMUs resulted in the largest correlations if all combinations were significantly different from the motion capture. No corrections were made for multiple comparisons so that we could detect smaller differences between IMU combinations.

Data are presented as mean ± standard deviation. Numbers are rounded to two decimal places (unless otherwise specified). A complete table of measures associated with each different combination of IMU placements can be found in the supplementary materials (S1 File).

## Results

### Sway characteristics

From the *CoM*_*FP*_, we computed the maximum sway range for the narrow stance (AP 25.18±9.48 mm; ML: 21.74±6.91 mm), hip-width stance (AP: 22.75±8.63 mm; ML: 10.77±5.67 mm), and shoulder-width stance (AP: 21.34±8.27 mm; ML: 8.13±4.11 mm). We also calculated the root mean square (RMS) sway for narrow stance (AP: 4.86±2.09 mm; ML: 4.21±1.49 mm), hip-width stance (AP: 4.33±1.51mm; ML: 2.04±1.20 mm), and shoulder-width stance (AP: 4.11±1.68 mm; ML: 1.69±0.85 mm).

### CoM estimation with back IMU

**Fig 2:**
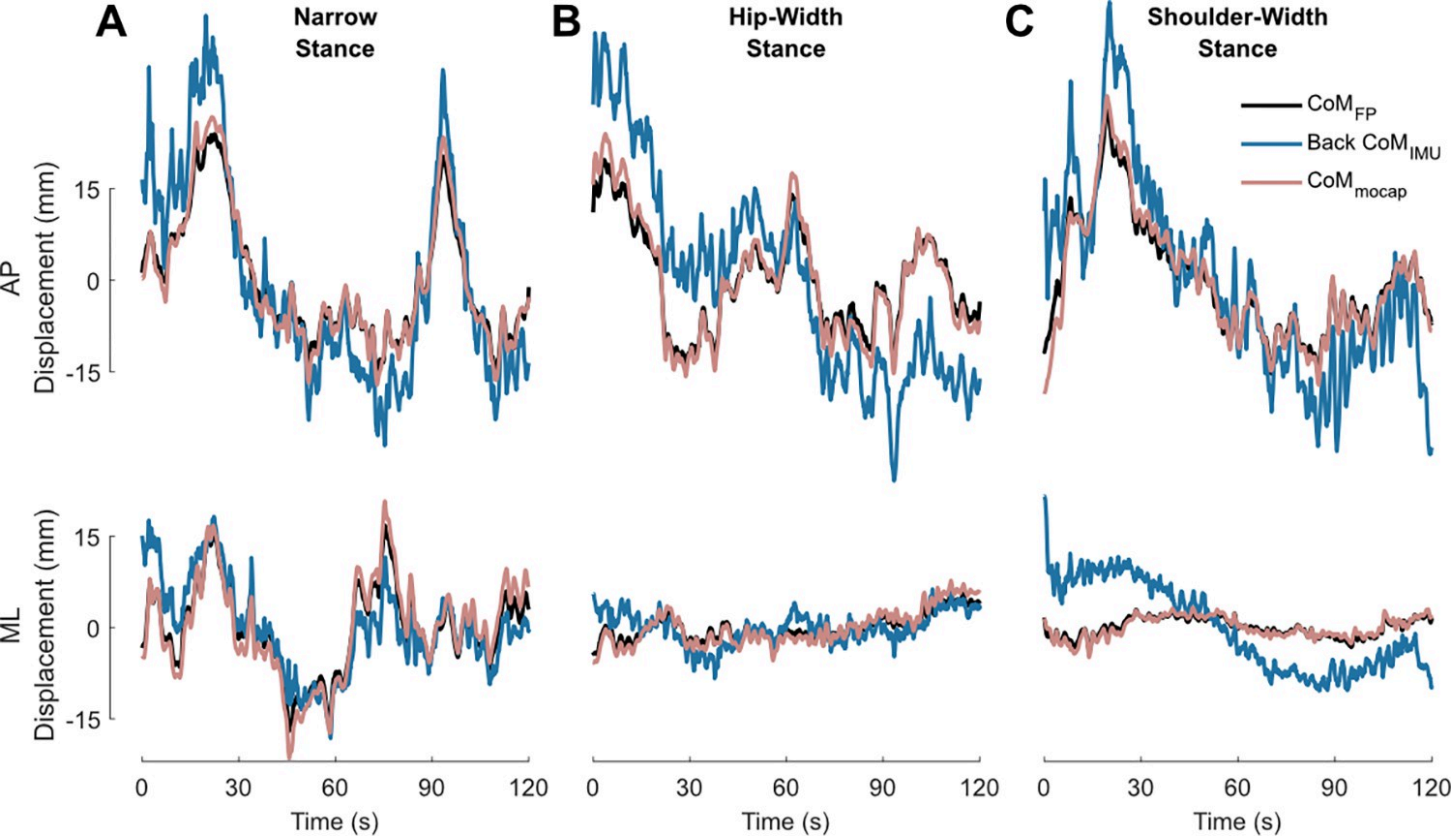
Sway estimation from force platform, motion caption, and single inertial measurement unit. Example trial time series from shoulder-width (A), hip-width (B), and narrow (C) stance comparing the centre of mass displacement estimation from the force platform (*CoM*_*FP*_), the linear displacement of the motion capture rigid body placed on the low back (*CoM*_*mocap*_), and a one-segment biomechanical model using the back inertial measurement unit orientation estimate (Back *CoM*_*IMU*_) in both anteroposterior (AP; above) and mediolateral (ML; below) directions. mm: millimetres, s: seconds.

As standing balance is often modelled as a single joint inverted pendulum in the AP direction (see Fig 1B), we initially estimated the *CoM*_*IMU*_ with the back (L3) IMU only. This resulted in variable correlations with the *CoM*_*FP*_ in both AP (across all stance widths: r = 0.51±0.21) and ML (r = 0.78±0.14, 0.39±0.21 and 0.13±0.36 for narrow, hip, and shoulder stance widths, respectively) (Fig 2). Similarly, we observed variable root mean square error (RMSE) values (AP, across all stance widths: 7.86±3.23 mm; ML, narrow: 2.78±1.70 mm, hip: 2.70±1.16 mm, shoulder: 3.11±1.87 mm).

Given that whole-body CoM can be approximated by the linear displacement of the trunk segment [13], we observed strong correlations between the *CoM*_*mocap*_ and the *CoM*_*FP*_ in AP (all stances widths: r = 0.97±0.02) and ML (r = 0.98±0.02, 0.93±0.06 and 0.87±0.11 for narrow, hip, and shoulder stance widths, respectively). Correspondingly, RMSE values were also lower (AP, all stance widths: 1.33±0.41 mm; ML, narrow: 1.27±0.52 mm, hip: 0.90±0.41 mm, shoulder: 0.86±0.44 mm).

### IMU CoM estimation

For each stance width, we compared the *CoM*_*IMU*_−*CoM*_*FP*_ correlations with the *CoM*_*mocap*_−*CoM*_*FP*_ correlations (Table 1). In the hip- and shoulder-width stances, some combinations did not reach the r >0.8 threshold, thus comparisons were only made for conditions that met the threshold. In the AP direction, every *CoM*_*IMU*_ combination was significantly different from the *CoM*_*mocap*_ across stance widths. Consequently, we determined the minimal number of IMUs as the combination that resulted in the highest overall AP estimates.

**Table 1:** The chosen sensor placements for each number of inertial measurement units (IMUs) and across stance widths.

	# IMUs	1	2	3	4	5	
**Narrow Stance**	**Total IMU Combo**	**Thigh**	**Left Shank** **& Thigh**	**Both Shanks** **& Thigh**	**Both Shanks,** **Thigh & Back**	**Both Shanks** **Thigh, Back** **& Sternum**	*Motion Capture*
	**AP IMU Combo**	Thigh	Left Shank Thigh	Both Shanks Thigh*	Both Shanks Thigh	Both Shanks Thigh	
	**Correlation**	0.87 (0.08)	0.90 (0.05)	0.92 (0.04)	0.92 (0.04)	0.92 (0.04)	*0*.*98 (0*.*02)*
	**Mocap Comparison**	t_15_ = 8.022 (p < .001)	t_15_ = 8.285 (p < .001)	t_15_ = 6.791 (p < .001)			
	**Pairwise Comparisons**		t_15_ = 1.679 (p = .057)	t_15_ = 2.095 (p = .027)				
	**ML IMU Combo**	Thigh	Left Shank Thigh*	Both Shanks*	Both Shanks Back*	Both Shanks Back Sternum*	
	**Correlation**	0.82 (0.15)	0.93 (0.06)	0.96 (0.04)	0.97 (0.02)	0.97 (0.03)	*0*.*98 (0*.*02)*
	**Mocap Comparison**	t_15_ = 8.638 (p < .001)	t_15_ = 5.420 (p < .001)	t_15_ = 2.724 (p = .008)	t_15_ = 1.138 (p = 0.136)	t_15_ = 0.653 (p = .262)	
	**Pairwise Comparisons**		t_15_ = 4.438 (p < .001)	t_15_ = 3.843 (p < .001)	t_15_ = 3.509 (p = .002)	t_15_ = 2.528 (p = .012)		
**Hip-Width Stance**	**Total IMU Combo**	N/A	N/A	**Both Shanks** **& Thigh**	**Both Shanks,** **Thigh & Back**	**Both Shanks** **Thigh, Back** **& Sternum**	
	**AP IMU Combo**	N/A	N/A	Both Shanks Thigh	Both Shanks Thigh	Both Shanks Thigh	
	**Correlation**			0.93 (0.04)	0.93 (0.04)	0.93 (0.04)	*0*.*96 (0*.*02)*
	**Mocap Comparison**			t_15_ = 5.284 (p < .001)			
	**Pairwise Comparisons**							
	**ML IMU Combo**	N/A	N/A	Both Shanks	Both Shanks Back*	Both Shanks Back Sternum*	
	**Correlation**			0.90 (0.06)	0.92 (0.06)	0.93 (0.05)	*0*.*93 (0*.*06)*
	**Mocap Comparison**			t_15_ = 1.870 (p = .041)	t_15_ = 0.521 (p = .305)	t_15_ = -0.148 (p = .558)	
	**Pairwise Comparisons**				t_15_ = 2.421 (p = .014)	t_15_ = 4.920 (p < .001)		
**Shoulder-Width Stance**	**Total IMU Combo**	N/A	**Both Shanks**	**Both Shanks** **& Thigh**	**Both Shanks,** **Thigh & Back**	**Both Shanks** **Thigh, Back** **& Sternum**	
	**AP IMU Combo**	N/A	Both Shanks	Both Shanks Thigh*	Both Shanks Thigh	Both Shanks Thigh	
	**Correlation**		0.80 (0.15)	0.93 (0.03)	0.93 (0.03)	0.93 (0.03)	*0*.*97 (0*.*02)*
	**Mocap Comparison**		t_15_ = 10.249 (p < .001)	t_15_ = 4.447 (p < .001)			
	**Pairwise Comparisons**			t_15_ = 5.762 (p < .001)				
	**ML IMU Combo**		Both Shanks	Both Shanks	Both Shanks Back*	Both Shanks Back Sternum*	
	**Correlation**		0.83 (0.13)	0.83 (0.13)	0.86 (0.13)	0.88 (0.12)	*0*.*87 (0*.*11)*
	**Mocap Comparison**		t_15_ = 1.593 (p = .066)		t_15_ = 0.146 (p = .443)	t_15_ = -0.471 (p = .678)	
	**Pairwise Comparisons**				t_15_ = 2.551 (p = .011)	t_15_ = 3.755 (p < .001)		

Pearson’s correlation coefficients are presented as mean (standard deviation). Underlined typeface indicates no significant difference from the centre of mass estimated with the motion capture data. *Indicates a significant difference from the previous IMU combination. AP: anteroposterior; ML: mediolateral.

**Fig 3:**
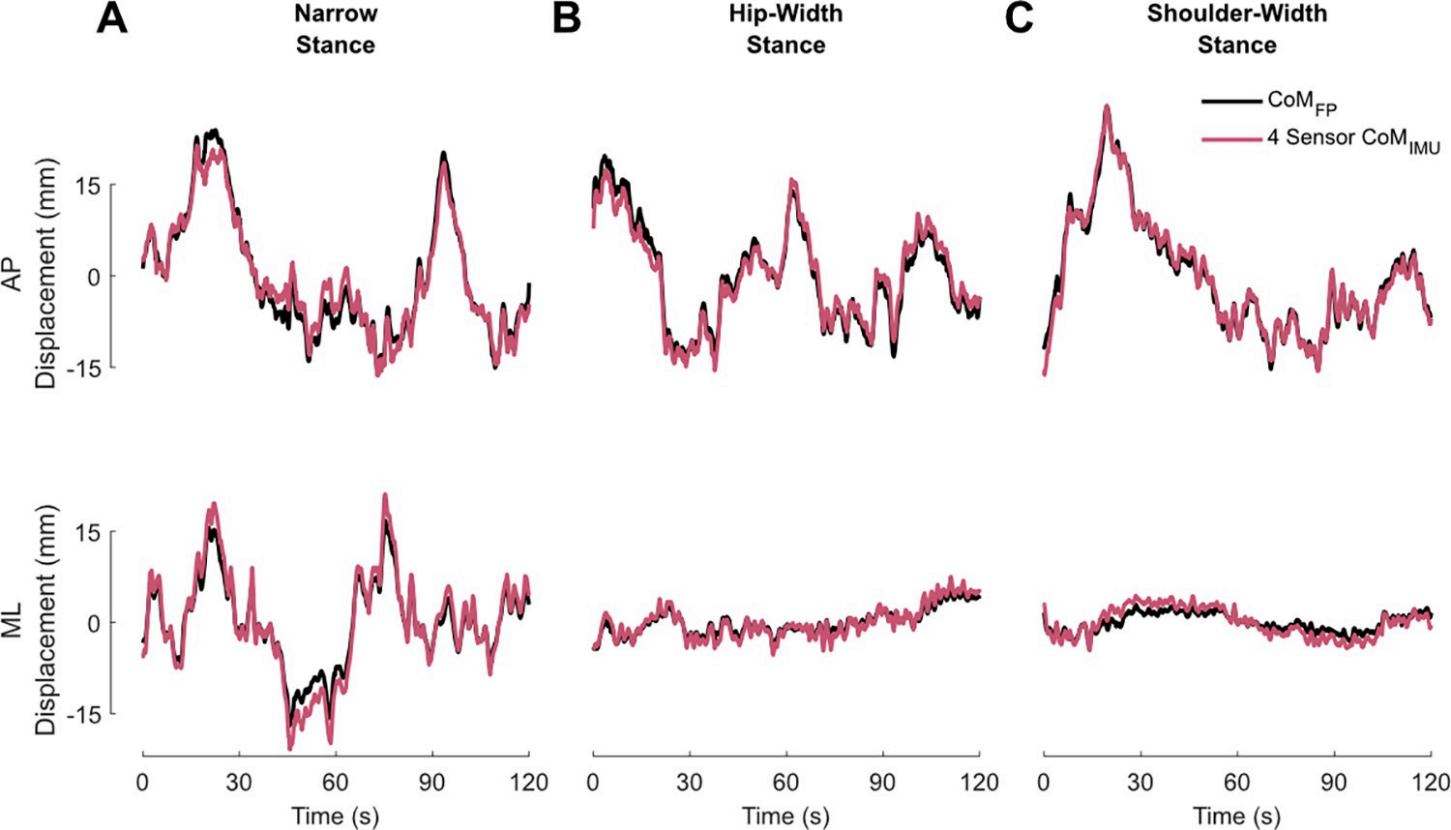
Sway estimation from force platform and four inertial measurement units. Example trial time series from narrow (A), hip-width (B), and shoulder-width (C) stance in both anteroposterior (AP; above) and mediolateral (ML; below) directions comparing the centre of mass displacement estimation from the force platform (*CoM*_*FP*_; black) and the centre of mass estimates using the recommended four inertial measurement units (IMUs; 4 Sensor *CoM*_*IMU*_; pink): both shanks, the right thigh, and the back. In the AP direction, the IMUs on the shanks and the thigh are used in the estimation algorithm. In ML, the IMUs on the shanks and back are used. mm: millimetres, s: seconds.

**Fig 4:**
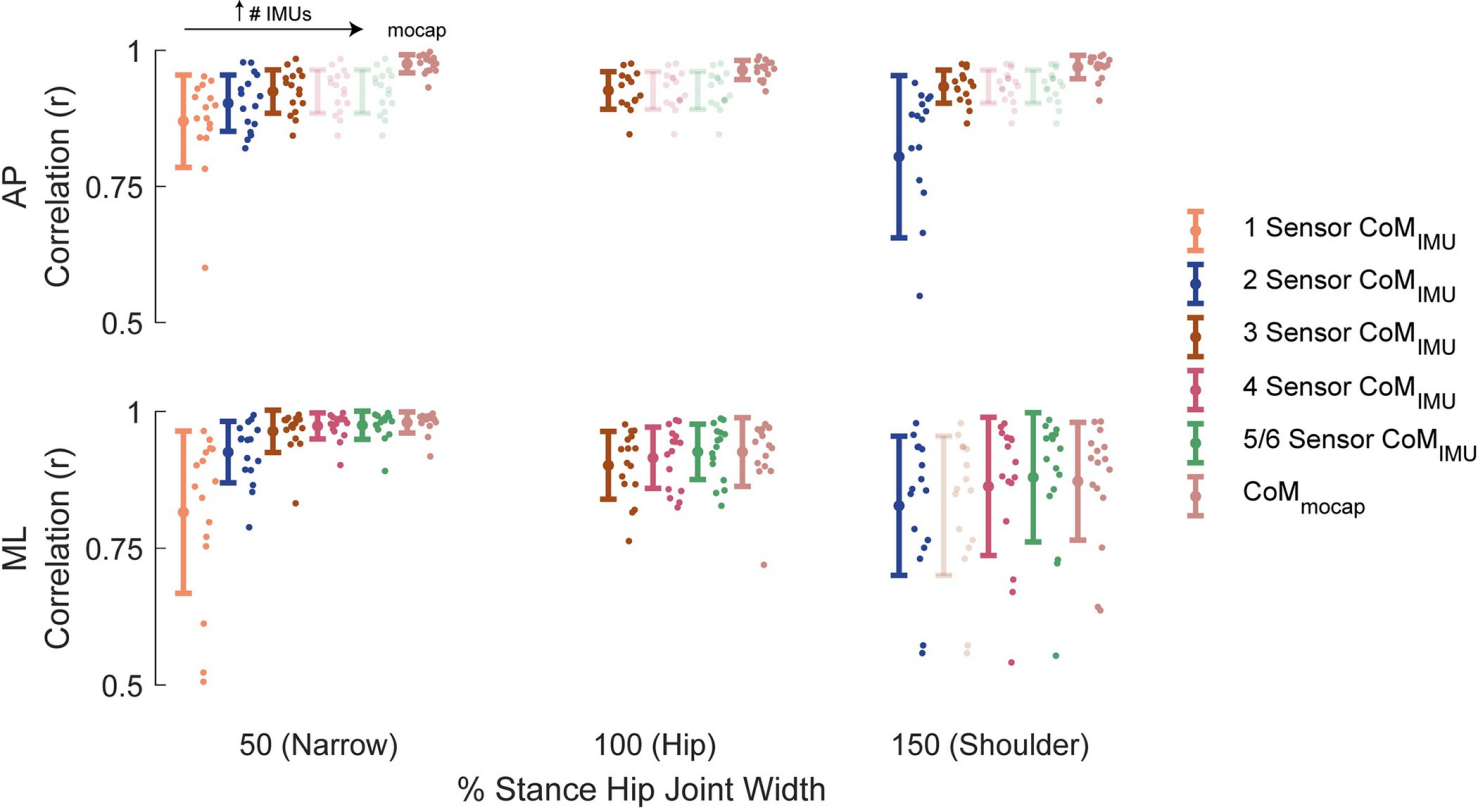
Comparison of number of inertial measurement units for sway estimation. Pearson’s correlation coefficients between the force platform centre of mass estimate and the motion capture linear displacement (*CoM*_*mocap*_; light red) or inertial measurement unit (IMU) centre of mass estimates (*CoM*_*IMU*_) in both anteroposterior (AP; above) and mediolateral (ML; below) directions. The following IMU set-ups are included: one IMU (1 Sensor *CoM*_*IMU*_; orange), two IMUs (2 Sensor *CoM*_*IMU*_; blue), up to three IMUs (3 Sensor *CoM*_*IMU*_; brown), up to four IMUs (4 Sensor *CoM*_*IMU*_; pink), and up to five or six IMUs (5/6 Sensor *CoM*_*IMU*_; green). Some IMU set-ups not included are because none of the combinations met the r > 0.8 threshold for both the AP and ML directions. Faded out data indicate that the sensor set-up is the same as the previous set-up. Markers with error bars illustrate the between participants mean ± standard deviation while markers show individual participant means (n = 16). The IMU set-ups are as follows: one IMU (Narrow–AP and ML: Thigh), two IMUs (Narrow–AP and ML: Opposite Shank & Thigh), up to three IMUs (shoulder-width–AP and ML: Both Shanks & Back; hip-width–AP: Both Shanks & Thigh, ML: Both Shanks; narrow–AP: Both Shanks & Thigh, ML: Both Shanks), up to four IMUs (all stance widths–AP: Both Shanks & Thigh, ML: Both Shanks & Back), and up to five or six IMUs (green; all stance widths–AP: Both Shanks & Thigh, ML: Both Shanks, Back, & Sternum).

*Narrow width stance*: the minimal number of IMUs required to estimate CoM sway was four: an IMU on both shanks, right thigh, and back. To determine the AP *CoM*_*IMU*_, the IMUs on both shanks and the thigh contributed to the estimate (r = 0.92±0.04; RMSE: 2.39±2.08 mm). For ML, the shank IMUs and the IMU on the back were included in the algorithm (r = 0.9735±0.02; RMSE: 1.16±0.77 mm) (Figs 3A and 4). Adding an additional IMU on the sternum for the ML estimation (increasing the total number of IMUs to five) significantly improved the ML *CoM*_*IMU*_ (r = 0.9748±0.03; RMSE: 1.12±0.78 mm).

*Hip-width stance*: the minimal number of total IMUs was again four: an IMU on both shanks, right thigh, and back. In the AP direction, the IMUs on both shanks and the thigh were required (r = 0.93±0.04; RMSE: 2.00±1.18 mm). For the ML *CoM*_*IMU*_, the IMUs on both shanks and the back were used (r = 0.92±0.06; RMSE: 0.92±0.70 mm) (Figs 3B and 4). By increasing the number of IMUs to five (adding sternum IMU) to the ML estimation, the ML *CoM*_*IMU*_ correlations significantly increased (r = 0.93±0.05; RMSE: 0.88±0.70 mm).

*Shoulder-width stance*: three total IMUs were required to obtain CoM estimates that did not differ from those estimated with motion capture: IMUs on both shanks and right thigh. For the AP *CoM*_*IMU*_, the IMUs on the shanks and the thigh were included (r = 0.93±0.03; RMSE: 1.95±1.66 mm). To determine the ML *CoM*_*IMU*_, only the IMUs on the shanks were included (r = 0.83±0.13; RMSE: 1.39±1.46 mm) (Figs 3C and 4). Adding an IMU on the back (four total IMUs) improved the ML*CoM*_*IMU*_ estimation to r = 0.86±0.13 (using both shanks and back; RMSE: 0.92±1.00 mm). Furthermore, including another IMU, on the sternum (increasing the total number to five) also significantly increased the ML *CoM*_*IMU*_ correlations (r = 0.88±0.12; RMSE: 0.91±1.00 mm).

## Discussion

The goal of this study was to determine the minimal number of wearable IMUs to adequately estimate CoM sway during quiet standing in both AP and ML across multiple stance widths. To do this, we developed an algorithm to estimate CoM sway using up to six IMUs. We found that IMUs were only needed on the lower limbs for AP CoM estimation and that a single IMU on the back was insufficient. Alternatively, IMUs were needed on the lower and upper body for ML CoM estimation. Across narrow, hip and shoulder stance widths, we found that four total IMUs (both shanks, right thigh, and back) were required to simultaneously estimate both AP and ML CoM similarly (with correlations >0.8 and RMSE <2.5 mm) to the *CoM*_*mocap*_. The IMUs on both shanks and the right thigh were used to estimate AP *CoM*_*IMU*_ while the IMUs on the shanks and the back contributed to ML *CoM*_*IMU*_ estimates. Furthermore, we found that as stance width increased, the accuracy and reliability of the ML *CoM*_*IMU*_ decreased across all different IMU combinations, but including more IMUs can improve the estimates.

### Single IMU CoM estimation

We first estimated *CoM*_*IMU*_ using a single IMU on the back based on a one-segment inverted pendulum model [13, 23, 28]. Contrary to our hypothesis, correlations of single IMU CoM estimation with the *CoM*_*FP*_ were poor in both AP and ML directions. While previous authors have reported accurate estimation of centre of pressure (CoP) sway during quiet standing with a single IMU placed on the back [19], we were unable to replicate this result with our CoM estimation. These differences persisted even when a larger 24x22cm rigid body or shorter sampling duration were used (see S1 File). We further validated with the motion capture data that these differences were not due to errors in the orientation estimation (see S1 File). Thus, these poor correlations are likely because local back orientation does not reflect the overall body sway during quiet standing.

### Multi-IMU CoM estimation

Given the shortcomings of the single IMU set-ups, we developed an algorithm to estimate whole-body CoM sway using a multi-segment biomechanical model. We found that for all stance widths, four IMUs were necessary to properly estimate CoM sway in AP and ML directions. An inverse dynamics approach with four IMUs has previously been used to estimate CoP sway during quiet standing [20]; however, this requires a fixed number of IMUs whereas our approach can provide greater flexibility to chose IMU placements depending on the use-case given our algorithm is robust to different combinations of IMUs. Another group of researcher used a multi-segment model to estimate CoM [29]. This approach also involved a fixed number of IMUs (seven) and yielded lower mean correlation values (AP: 0.58 vs 0.93; ML: 0.78 vs 0.92) and larger the mean RMSE values (AP: 6.5 mm vs 2 mm; ML: 2.5 mm vs 0.92 mm) than our hip width stance results. These differences are likely caused by differences in the biomechanical models. Notably, we estimated the CoM based on the CoM from segments across the entire body, whereas the previous study modelled only the lower body to estimate the movement of the pelvis, which they assumed to be the whole-body CoM location.

For AP quiet standing sway, we found that IMUs located on the shanks and right thigh provided good estimates of CoM. Hence, AP *CoM*_*IMU*_ is effectively driven by lower limb angle estimates, as previously described [13]. Additional IMUs may slightly worsen the AP estimates because the local segment orientations further up the body may not reflect the whole-body CoM sway. Alternatively, during ML sway, we found that IMUs were needed on the shanks and back for the highest correlations between *CoM*_*IMU*_ and *CoM*_*FP*_. This fits with the quadrilateral model often used to model ML sway [10, 15], given the possible counter-rotations between the upper and lower body segments.

### Stance width differences

For ML CoM sway, we observed improved *CoM*_*mocap*_ and *CoM*_*IMU*_ estimates across all combinations as stance width decreased and whole-body sway increased. These observations are likely related: the ratio between the signal amplitude and the noise from measurements and estimation (related to the biomechanical models) decreases as stance width increases, corrupting the whole-body displacement estimates. From narrow stance to hip- and shoulder-width stance, the CoM sway range and RMS decreased by 50–63%, which illustrates the decreased ML sway amplitude at wider stances. This also poses a limitation for the *CoM*_*IMU*_ algorithm, as ML CoM estimates worsen for wider compared to narrow stance conditions. These changes in ML CoM estimation across stance widths could also be due to the changing biomechanics during increasing stance width, where models suggest an increased counter-rotation at the hips in quiet standing [30–32].

### Recommendations

We recommend that four total IMUs (both shanks, thigh, and back) should be used to properly estimate AP and ML CoM during quiet standing across stance widths. For the AP direction, IMUs on the shanks and thigh should be used in the CoM estimation algorithm, while IMUs on the shanks and back should be used in the algorithm for ML CoM estimation. The flexibility of the algorithm allows users to add more IMUs if they are available.

### Limitations

A limitation of this study was the number and placement of the IMUs used to estimate whole-body CoM sway. We used the current IMU locations to characterize sway of the main segments (lower limbs, trunk, head) exhibiting relative motion during quiet standing, but the algorithm could be adjusted to account for IMUs on other segments using the same framework. Given the importance of the right thigh IMU in estimating AP CoM sway, adding an IMU to the left thigh may further improve the AP CoM estimation. Future work is also needed to improve ML CoM estimation at wider stance widths.

Another limitation was the whole-body CoM estimation method we used to validate the results. While previous work has supported the zero-point-to-zero-point integration method as an appropriate method to estimate whole body CoM compared to motion capture (< 1 mm RMSE) [27], there is evidence suggesting that low pass filtering of the centre of pressure may be preferable to estimate CoM from a force platform [33, 34]. To address this limitation, we compared the results when estimating CoM with our approach involving zero-point-to-zero-point integration to a low-pass filter of the centre of pressure data. We did not find major differences between these methods (r ≤ 0.02 and RMSE ≤ 0.15 mm), and a comparison between the two is available in the supplementary materials (S1 File.

## Conclusion

Here, we validated a novel approach based on existing biomechanical models to estimate quiet standing CoM displacements using wearable IMUs. We determined a single sensor on a lower limb or back was inadequate to estimate both AP and ML CoM and that four total IMUs are required for acceptable estimates. Using this novel algorithm, researchers and clinicians can estimate whole-body CoM accurately during unperturbed quiet standing. This approach can improve the ecological validity of standing balance research and opens the possibility for assessing/monitoring patients with standing balance deficits.

## Supporting information

S1 File Supporting text for supplementary methodology and results. (PDF)
