# Supplementary material for: Estimating whole-body centre of mass sway during quiet standing with inertial measurement units
Source: PLoS One. 2025 Jan 13;20(1):e0315851. doi: 10.1371/journal.pone.0315851 (PMC11730423; doi:10.1371/journal.pone.0315851)
Supplement: S1 File — (PDF) [file pone.0315851.s001.pdf]

Methods

IMU Orientation

We determined the orientation of the IMU using a complementary filter [1]. This determines the tilt orientation of the IMU along the pitch (around Y-axis) and roll (around X-axis) axes with the following equation:

$$\boldsymbol{\theta_i = \left(\theta_{i-1} + \omega_{i,r} * dt\right) G + (acc_i)(1 - G)}$$

In this, $\boldsymbol{\theta_i}$ denotes the ith orientation estimate with respect to the vertical axis in one dimension. $\boldsymbol{\omega_{i,r}}$ corresponds to the rotated angular velocity around the identical axis, obtained from the gyroscope. The time step between samples (4ms) is represented with $\boldsymbol{dt}$, $\boldsymbol{G}$ is the weighting factor between the gyroscope and accelerometer estimates (set to 0.995), and $\boldsymbol{acc_i}$ represents the accelerometer orientation estimate in the same dimension. In cases where the net acceleration deviated by 10% or more from 9.81 m/s^2, only gyroscope information was utilized for orientation estimation at that specific sample point.

To calculate the pitch and roll accelerometer orientation estimates, the following equations were used:

$$\boldsymbol{pitch = acc_i = atan2d(\ddot{x}_i, \ddot{z}_i)}$$

$$\boldsymbol{roll = acc_i = -\,atan2d\left(\ddot{y}_i, \sqrt{\ddot{x}_i^2 + \ddot{z}_i^2}\right)}$$

Additionally, the raw angular velocities ($\boldsymbol{\omega_i}$) were rotated using the previous estimated rotation matrix ($\boldsymbol{R}$) prior to the complementary filter integration:

$$\boldsymbol{\omega_{i,r} = \omega_i R_{i-1}}$$

Results

Orientation Estimation

To estimate the whole-body CoM, we first needed to determine how accurately we could estimate body segment orientations using the IMU data. To determine the orientation of the rigid body, we calculated a 3×3 rotation matrix to define a frame from the three motion capture markers. To define the frame, we calculated the frame Y-axis as the directed between the lower two markers on the triangular rigid body from left to right (Fig S1). The frame Z-axis was defined perpendicular to the Y-axis and away from the upper motion capture marker on the apex of the rigid body. Finally, the frame X-axis was defined as the cross product of the Z-axis and Y-axis. We then used Euler angles to specify the rotation of the frame in space as the pitch and roll of the motion capture rigid bodies [2]. Offsets were removed to facilitate comparisons between the orientation estimates. We compared motion capture data from a three-marker rigid body that was fixed to the IMU placed on the low back to validate the IMU orientation estimation in both pitch (around Y-axis) and roll (around X-axis). In the pitch direction, the average range of orientation estimated from the motion capture was 3.07° ± 1.10° SD across all conditions (see sample single participant data in Figure S1A). In the roll axis, the orientation range was similar between shoulder-width stance (0.92° ± 0.33° SD) and hip-width stance (0.91° ± 0.26° SD) but increased with narrow stance (1.47° ± 0.49° SD). The orientation estimates between the motion capture and IMU were highly correlated in both pitch ($r = 0.98 \pm 0.04$ SD) and roll ($r = 0.97 \pm 0.03$ SD) (Fig S1B). Correspondingly, the root mean square error (RMSE) between the two estimates were low: 0.09º ± 0.04 SD in pitch and 0.05º ± 0.02 SD in roll (Fig S1C).

Comparison of Force Platform CoM Estimation Methods

To confirm that the chosen method to estimate the whole body CoM displacement from the force platform (the zero-point-to-zero-point double integration method [3]) did not affect our results, we repeated our analyses using an alternative method based on low pass filtering of the centre of pressure [4]. We did not find any major differences between the methods from the results presented in the main manuscript (difference in r ≤ 0.02 and difference in RMSE ≤ 0.15 mm; Table S1).

Low Back Rigid Body CoM Estimation

Since previous studies [5,6] have placed the IMU on a large rigid body wrapped around the low back – possibly to minimize any effects of small changes to the local back orientation – we collected the same protocol with the low back sensor placed on a large rigid body (24 cm × 22 cm) that was strapped to the participant at L3 with an elastic waistband. A separate calibration was performed for these rigid body trials and the order of stance widths was also randomized. The rigid body and non-rigid body trials were grouped for logistical simplicity and the order was randomized between participants. However, we did not find any differences between the trials with the large rigid body and without (see Tables S2 and S3).

Trial Duration Effects

Since a previous study [5] that validated the use of a single low back sensor used only 30 seconds of recording time, we wanted to determine if this duration difference was responsible for the large differences in correlations between the low back CoM_{IMU} and CoM_{FP} in this study and

the previous one. The trial duration of the previous study is much shorter than what was done in this experiment (30 seconds vs 120 seconds); however, when we trim our data to match this, the mean correlation coefficients across the IMU combinations presented in the results only change by -0.1 to 0.03. Thus, it is unlikely that the difference in trial duration is responsible for this difference in results as well.

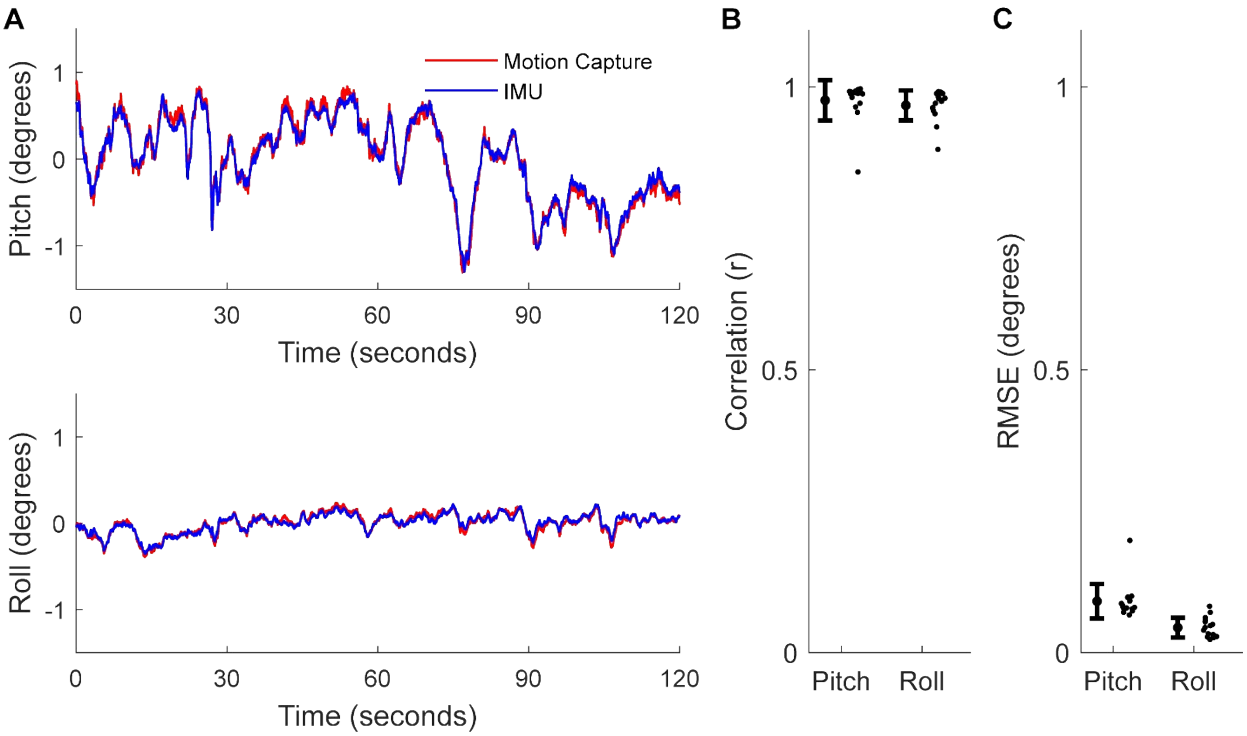

Fig S1. Orientation estimation results and comparison. A) Example trial time series comparing the orientation estimation from the three-marker motion capture rigid body placed on the low back ("Motion Capture"; red) and the inertial measurement unit with a complementary filter ("IMU"; blue) in pitch (rotation around Y-axis; above) and roll (rotation around X-axis; below). B) Pearson's correlation coefficients between the pitch and roll predicted from the three-marker motion capture rigid body placed on the low back and the inertial measurement unit at the same location with a complementary filter. Error bar: across subject mean ± standard deviation. Markers: individual subject means. C) Root mean square error between the pitch and roll predicted from the inertial measurement unit on the back with a complementary filter and the motion capture rigid body. Error bar: across subject mean ± standard deviation. Markers: individual subject means averaged across conditions (n = 16).

			Narrow		Hip		Shoulder	
			GLP	LPF	GLP	LPF	GLP	LPF
**Mocap CoM**	AP	r	0.98±0.02	0.96±0.02	0.96±0.02	0.95±0.02	0.97±0.02	0.96±0.02
		RMSE	1.34±0.45	1.45±0.44	1.42±0.42	1.50±0.42	1.24±0.37	1.33±0.36
	ML	r	0.98±0.02	0.97±0.02	0.93±0.06	0.91±0.07	0.87±0.11	0.86±0.10
		RMSE	1.27±0.52	1.35±0.50	0.90±0.41	0.95±0.40	0.86±0.44	0.92±0.42
**Back IMU**	AP	r	0.54±0.28	0.53±0.27	0.48±0.18	0.47±0.18	0.54±0.18	0.54±0.17
		RMSE	7.63±3.30	7.69±3.31	8.35±3.29	8.38±3.29	7.60±3.25	7.63±3.25
	ML	r	0.78±0.14	0.78±0.14	0.39±0.21	0.38±0.20	0.13±0.36	0.13±0.35
		RMSE	2.78±1.70	2.88±1.70	2.70±1.16	2.76±1.18	3.11±1.87	3.16±1.88
**Both Shanks IMUs**	AP	r	0.75±0.18	0.75±0.17	0.75±0.15	0.74±0.15	0.80±0.15	0.80±0.15
		RMSE	3.85±2.58	3.94±2.57	3.61±2.34	3.73±2.33	2.69±1.58	2.82±1.59
	ML	r	0.96±0.04	0.95±0.04	0.90±0.06	0.89±0.06	0.83±0.13	0.81±0.12
		RMSE	1.54±0.97	1.62±0.97	1.20±0.88	1.23±0.89	1.39±1.46	1.42±1.46
**Both Shanks + Thigh IMUs**	AP	r	0.92±0.04	0.92±0.04	0.93±0.04	0.92±0.03	0.93±0.03	0.92±0.03
		RMSE	2.39±2.08	2.49±2.05	2.00±1.18	2.08±1.14	1.95±1.66	2.04±1.62
	ML	r	0.95±0.03	0.94±0.04	0.86±0.1	0.85±0.10	0.79±0.15	0.79±0.14
		RMSE	1.45±0.89	1.56±0.90	1.25±0.72	1.28±0.72	1.3±1.08	1.33±1.07
**Both Shanks + Back IMUs**	AP	r	0.84±0.12	0.83±0.12	0.8±0.12	0.8±0.12	0.86±0.09	0.85±0.09
		RMSE	2.91±1.61	3.04±1.59	2.94±1.58	3.07±1.58	2.25±1.17	2.40±1.17
	ML	r	0.97±0.02	0.96±0.02	0.92±0.06	0.90±0.06	0.86±0.13	0.84±0.12
		RMSE	1.16±0.77	1.29±0.77	0.92±0.70	0.98±0.71	0.92±1.00	1.00±0.99
**Both Shanks, Back + Sternum IMUs**	AP	r	0.83±0.12	0.83±0.12	0.82±0.10	0.81±0.10	0.86±0.10	0.85±0.09
		RMSE	2.88±1.64	3.00±1.63	2.81±1.53	2.94±1.53	2.20±1.13	2.34±1.13
	ML	r	0.97±0.03	0.96±0.03	0.93±0.05	0.91±0.05	0.88±0.12	0.86±0.12
		RMSE	1.12±0.78	1.27±0.77	0.88±0.70	0.95±0.71	0.91±1.00	0.98±1.00

Table S1. Comparison between the zero-point-to-zero-point integration (GLP) and low pass filter (LPF) force plate centre of mass (CoM) estimation methods for validating the inertial measurement unit (IMU) CoM estimation methods. Pearson correlation coefficient (r) and root mean square error (RMSE) values are presented between the force platform methods (GLP and LPF) and the IMU/motion capture methods in both anteroposterior (AP) and mediolateral (ML) directions. Comparisons located in the grey boxes contain comparisons not included in the main manuscript (i.e., because the combination of IMUs and directions were not recommended). However, we have included them here to further illustrate that only minor differences exist between the GLP and LPF methods.

	Sensor Placement	Direction	Shoulder-Width Stance		Hip-Width Stance		Narrow-Width Stance	
			Correlation (r) Mean (SD)	***RMSE (mm)*** Mean (SD)	***Correlation (r)*** Mean (SD)	***RMSE (mm)*** Mean (SD)	***Correlation (r)*** Mean (SD)	***RMSE (mm)*** Mean (SD)
**1 IMU**	Shank	AP	0.69 (0.24)	3.39 (2.20)	0.65 (0.21)	4.51 (2.67)	0.62 (0.21)	5.06 (2.96)
		ML	0.71 (0.18)	2.31 (2.71)	0.79 (0.18)	2.19 (2.42)	0.87 (0.20)	2.68 (2.07)
	Right Thigh	AP	0.88 (0.09)	3.69 (3.66)	0.84 (0.10)	3.90 (2.50)	0.87 (0.08)	4.34 (4.07)
		ML	0.57 (0.26)	2.02 (1.18)	0.62 (0.29)	2.57 (2.02)	0.81 (0.15)	2.66 (1.61)
	Back	AP	0.54 (0.18)	7.60 (3.25)	0.48 (0.18)	8.35 (3.29)	0.54 (0.28)	7.63 (3.30)
		ML	0.13 (0.36)	3.11 (1.87)	0.39 (0.21)	2.70 (1.16)	0.78 (0.14)	2.78 (1.70)
	Sternum	AP	0.24 (0.19)	13.23 (4.26)	0.22 (0.20)	15.35 (5.98)	0.22 (0.23)	13.33 (4.93)
		ML	0.31 (0.25)	4.66 (1.49)	0.30 (0.21)	4.79 (2.01)	0.48 (0.16)	5.12 (1.65)
	Head	AP	0.22 (0.26)	17.96 (10.53)	0.21 (0.24)	21.10 (12.34)	0.24 (0.33)	17.71 (9.07)
		ML	0.15 (0.27)	8.56 (3.52)	0.16 (0.29)	8.88 (4.61)	0.26 (0.24)	9.00 (4.46)
**2 IMUs**	Left Shank Right Shank	AP	0.80 (0.15)	2.69 (1.58)	0.75 (0.15)	3.61 (2.34)	0.75 (0.18)	3.85 (2.58)
		ML	0.83 (0.13)	1.39 (1.46)	0.90 (0.06)	1.20 (0.88)	0.96 (0.04)	1.54 (0.97)
	Right Shank Right Thigh	AP	0.91 (0.05)	2.14 (1.65)	0.91 (0.05)	2.21 (1.41)	0.90 (0.05)	2.64 (2.13)
		ML	0.68 (0.19)	1.85 (1.42)	0.72 (0.21)	2.08 (1.61)	0.88 (0.11)	2.16 (1.37)
	Left Shank Right Thigh	AP	0.92 (0.04)	2.11 (1.74)	0.91 (0.04)	2.24 (1.26)	0.90 (0.05)	2.75 (2.19)
		ML	0.71 (0.21)	1.50 (1.10)	0.78 (0.15)	1.62 (0.98)	0.92 (0.06)	1.71 (1.16)
	Right Shank Back	AP	0.81 (0.13)	2.64 (1.39)	0.74 (0.16)	3.50 (1.90)	0.74 (0.13)	3.78 (1.81)
		ML	0.75 (0.17)	1.66 (1.87)	0.80 (0.18)	1.71 (1.92)	0.90 (0.16)	2.00 (1.55)
	Right Shank Sternum	AP	0.61 (0.19)	4.09 (2.02)	0.60 (0.15)	4.83 (1.99)	0.59 (0.21)	4.97 (2.82)
		ML	0.74 (0.17)	1.94 (1.93)	0.77 (0.18)	1.93 (1.87)	0.88 (0.17)	2.20 (1.63)
	Right Shank Head	AP	0.70 (0.24)	3.40 (2.32)	0.66 (0.20)	4.45 (2.68)	0.63 (0.22)	4.96 (2.92)
		ML	0.73 (0.18)	2.20 (2.58)	0.79 (0.18)	2.10 (2.37)	0.87 (0.20)	2.55 (2.05)
	Right Thigh Back	AP	0.87 (0.09)	3.53 (2.74)	0.82 (0.10)	3.75 (1.91)	0.86 (0.09)	4.08 (3.24)
		ML	0.57 (0.27)	1.71 (0.89)	0.65 (0.28)	2.13 (1.50)	0.86 (0.12)	2.16 (1.33)
	Right Thigh Sternum	AP	0.74 (0.13)	4.28 (2.47)	0.71 (0.10)	4.91 (2.00)	0.76 (0.11)	4.55 (2.65)
		ML	0.63 (0.22)	1.76 (0.87)	0.63 (0.27)	2.29 (1.59)	0.83 (0.12)	2.35 (1.25)
	Right Thigh Head	AP	0.88 (0.08)	3.55 (3.47)	0.83 (0.11)	3.87 (2.37)	0.87 (0.09)	4.16 (3.79)
		ML	0.58 (0.26)	1.95 (1.11)	0.63 (0.29)	2.49 (1.92)	0.82 (0.14)	2.59 (1.57)
	Back Sternum	AP	0.55 (0.17)	7.31 (2.99)	0.48 (0.18)	8.02 (3.05)	0.54 (0.27)	7.36 (3.07)
		ML	0.15 (0.37)	3.06 (1.83)	0.40 (0.21)	2.66 (1.15)	0.78 (0.14)	2.75 (1.65)
	Back Head	AP	0.55 (0.18)	7.50 (3.20)	0.48 (0.18)	8.28 (3.26)	0.54 (0.28)	7.54 (3.25)
		ML	0.13 (0.36)	3.10 (1.86)	0.39 (0.21)	2.70 (1.17)	0.78 (0.14)	2.77 (1.68)
	Sternum Head	AP	0.24 (0.19)	13.16 (4.23)	0.22 (0.20)	15.24 (5.92)	0.22 (0.23)	13.24 (4.91)
		ML	0.31 (0.25)	4.66 (1.49)	0.30 (0.21)	4.78 (2.00)	0.48 (0.16)	5.13 (1.66)

	Sensor Placement	Direction	Shoulder-Width Stance		Hip-Width Stance		Narrow-Width Stance	
			Correlation (r) Mean (SD)	*RMSE (mm)* Mean (SD)	*Correlation (r)* Mean (SD)	*RMSE (mm)* Mean (SD)	*Correlation (r)* Mean (SD)	*RMSE (mm)* Mean (SD)
**3 IMUs**	Left Shank Right Shank Right Thigh	AP	0.93 (0.03)	1.95 (1.66)	0.93 (0.03)	2.00 (1.18)	0.92 (0.04)	2.39 (2.08)
		ML	0.79 (0.15)	1.30 (1.08)	0.86 (0.10)	1.25 (0.72)	0.95 (0.03)	1.45 (0.89)
	Left Shank Right Shank Back	AP	0.86 (0.09)	2.25 (1.17)	0.80 (0.12)	2.94 (1.58)	0.84 (0.12)	2.91 (1.61)
		ML	0.86 (0.13)	0.92 (1.00)	0.92 (0.06)	0.92 (0.70)	0.97 (0.02)	1.16 (0.77)
	Left Shank Right Shank Sternum	AP	0.65 (0.18)	3.78 (1.64)	0.64 (0.13)	4.40 (1.78)	0.67 (0.20)	4.15 (2.30)
		ML	0.83 (0.14)	1.31 (1.01)	0.86 (0.08)	1.25 (0.71)	0.95 (0.04)	1.39 (0.78)
	Left Shank Right Shank Head	AP	0.79 (0.18)	2.77 (1.75)	0.75 (0.14)	3.58 (2.33)	0.75 (0.19)	3.81 (2.49)
		ML	0.84 (0.13)	1.32 (1.39)	0.90 (0.07)	1.16 (0.84)	0.96 (0.05)	1.44 (0.96)
	Left Shank Right Thigh Back	AP	0.88 (0.07)	2.25 (1.00)	0.88 (0.07)	2.32 (0.95)	0.89 (0.07)	2.57 (1.48)
		ML	0.72 (0.23)	1.21 (0.93)	0.80 (0.14)	1.33 (0.71)	0.94 (0.04)	1.42 (1.01)
	Left Shank Right Thigh Sternum	AP	0.72 (0.15)	3.42 (1.13)	0.73 (0.11)	3.89 (1.52)	0.76 (0.12)	3.50 (1.26)
		ML	0.74 (0.18)	1.41 (0.82)	0.76 (0.15)	1.56 (0.84)	0.92 (0.05)	1.60 (0.95)
	Left Shank Right Thigh Head	AP	0.91 (0.06)	2.14 (1.57)	0.90 (0.05)	2.32 (1.20)	0.89 (0.06)	2.69 (1.89)
		ML	0.72 (0.21)	1.43 (1.02)	0.78 (0.16)	1.56 (0.90)	0.93 (0.06)	1.64 (1.11)
	Right Shank Right Thigh Back	AP	0.90 (0.05)	2.02 (0.89)	0.87 (0.09)	2.32 (1.04)	0.89 (0.05)	2.48 (1.25)
		ML	0.72 (0.16)	1.44 (1.05)	0.76 (0.19)	1.65 (1.28)	0.92 (0.07)	1.72 (1.06)
	Right Shank Right Thigh Sternum	AP	0.72 (0.16)	3.40 (1.28)	0.72 (0.12)	3.80 (1.62)	0.73 (0.13)	3.72 (1.52)
		ML	0.74 (0.16)	1.61 (1.17)	0.73 (0.18)	1.83 (1.33)	0.89 (0.10)	1.90 (1.15)
	Right Shank Right Thigh Head	AP	0.89 (0.08)	2.19 (1.49)	0.89 (0.06)	2.34 (1.44)	0.89 (0.06)	2.64 (1.92)
		ML	0.70 (0.19)	1.76 (1.38)	0.73 (0.22)	1.99 (1.57)	0.88 (0.12)	2.08 (1.38)
	Right Shank Back Sternum	AP	0.80 (0.13)	2.61 (1.43)	0.75 (0.13)	3.41 (1.82)	0.73 (0.14)	3.80 (1.93)
		ML	0.77 (0.17)	1.64 (1.90)	0.81 (0.18)	1.69 (1.92)	0.90 (0.16)	1.99 (1.57)
	Right Shank Back Head	AP	0.81 (0.13)	2.62 (1.41)	0.74 (0.17)	3.50 (1.92)	0.74 (0.13)	3.78 (1.84)
		ML	0.76 (0.17)	1.66 (1.88)	0.81 (0.18)	1.71 (1.93)	0.90 (0.16)	2.00 (1.56)
	Right Shank Sternum Head	AP	0.62 (0.19)	4.05 (2.04)	0.60 (0.14)	4.77 (2.01)	0.59 (0.21)	4.93 (2.82)
		ML	0.74 (0.17)	1.94 (1.94)	0.77 (0.18)	1.93 (1.88)	0.88 (0.18)	2.20 (1.64)
	Right Thigh Back Sternum	AP	0.87 (0.08)	3.37 (2.72)	0.83 (0.09)	3.59 (1.94)	0.86 (0.08)	3.90 (3.15)
		ML	0.60 (0.26)	1.66 (0.88)	0.66 (0.28)	2.11 (1.52)	0.86 (0.12)	2.15 (1.32)
	Right Thigh Back Head	AP	0.87 (0.09)	3.45 (2.73)	0.82 (0.10)	3.70 (1.90)	0.86 (0.09)	4.02 (3.21)
		ML	0.58 (0.27)	1.70 (0.89)	0.65 (0.28)	2.13 (1.50)	0.86 (0.12)	2.16 (1.33)
	Right Thigh Sternum Head	AP	0.75 (0.12)	4.21 (2.48)	0.72 (0.09)	4.81 (2.00)	0.77 (0.11)	4.47 (2.66)
		ML	0.63 (0.23)	1.77 (0.86)	0.63 (0.27)	2.29 (1.58)	0.83 (0.12)	2.36 (1.25)
	Back Sternum Head	AP	0.55 (0.17)	7.27 (2.99)	0.48 (0.18)	8.02 (3.07)	0.54 (0.27)	7.33 (3.07)
		ML	0.15 (0.37)	3.06 (1.83)	0.39 (0.21)	2.67 (1.17)	0.78 (0.14)	2.76 (1.65)

	Sensor Placement	Direction	Shoulder-Width Stance Correlation (r) Mean (SD)	Shoulder-Width Stance RMSE (mm) Mean (SD)	Hip-Width Stance Correlation (r) Mean (SD)	Hip-Width Stance RMSE (mm) Mean (SD)	Narrow-Width Stance Correlation (r) Mean (SD)	Narrow-Width Stance RMSE (mm) Mean (SD)
4 IMUs	Left Shank Right Shank Right Thigh Back	AP	0.91 (0.05)	1.97 (0.81)	0.89 (0.07)	2.11 (0.79)	0.92 (0.05)	2.20 (1.22)
		ML	0.84 (0.15)	0.91 (0.82)	0.89 (0.08)	0.96 (0.53)	0.97 (0.02)	1.12 (0.76)
	Left Shank Right Shank Right Thigh Sternum	AP	0.73 (0.15)	3.30 (1.15)	0.74 (0.12)	3.70 (1.49)	0.77 (0.13)	3.37 (1.34)
		ML	0.81 (0.13)	1.25 (0.80)	0.84 (0.09)	1.25 (0.64)	0.95 (0.03)	1.36 (0.74)
	Left Shank Right Shank Right Thigh Head	AP	0.91 (0.05)	2.01 (1.47)	0.91 (0.05)	2.11 (1.15)	0.91 (0.05)	2.35 (1.83)
		ML	0.81 (0.14)	1.23 (1.02)	0.86 (0.12)	1.20 (0.65)	0.96 (0.04)	1.37 (0.86)
	Left Shank Right Shank Back Sternum	AP	0.86 (0.10)	2.20 (1.13)	0.82 (0.10)	2.81 (1.53)	0.83 (0.12)	2.88 (1.64)
		ML	0.88 (0.12)	0.91 (1.00)	0.93 (0.05)	0.88 (0.70)	0.97 (0.03)	1.12 (0.78)
	Left Shank Right Shank Back Head	AP	0.86 (0.10)	2.22 (1.17)	0.81 (0.12)	2.92 (1.60)	0.84 (0.13)	2.89 (1.62)
		ML	0.86 (0.13)	0.91 (1.00)	0.92 (0.06)	0.91 (0.70)	0.97 (0.03)	1.15 (0.77)
	Left Shank Right Shank Sternum Head	AP	0.65 (0.17)	3.73 (1.64)	0.65 (0.12)	4.32 (1.78)	0.67 (0.19)	4.10 (2.30)
		ML	0.83 (0.14)	1.31 (1.01)	0.86 (0.08)	1.24 (0.71)	0.95 (0.05)	1.39 (0.79)
	Left Shank Right Thigh Back Sternum	AP	0.89 (0.05)	2.09 (0.90)	0.89 (0.05)	2.14 (0.90)	0.90 (0.06)	2.40 (1.33)
		ML	0.74 (0.22)	1.17 (0.91)	0.81 (0.14)	1.30 (0.73)	0.95 (0.04)	1.38 (0.99)
	Left Shank Right Thigh Back Head	AP	0.89 (0.06)	2.18 (0.99)	0.88 (0.06)	2.28 (0.91)	0.90 (0.06)	2.51 (1.44)
		ML	0.72 (0.23)	1.20 (0.92)	0.80 (0.14)	1.32 (0.71)	0.94 (0.04)	1.41 (1.00)
	Left Shank Right Thigh Sternum Head	AP	0.73 (0.14)	3.36 (1.12)	0.74 (0.11)	3.79 (1.49)	0.77 (0.11)	3.42 (1.24)
		ML	0.74 (0.18)	1.41 (0.82)	0.76 (0.15)	1.55 (0.82)	0.92 (0.05)	1.60 (0.95)
	Right Shank Right Thigh Back Sternum	AP	0.91 (0.04)	1.88 (0.82)	0.89 (0.06)	2.13 (0.98)	0.89 (0.04)	2.40 (1.17)
		ML	0.74 (0.16)	1.41 (1.07)	0.77 (0.19)	1.62 (1.30)	0.92 (0.08)	1.69 (1.08)
	Right Shank Right Thigh Back Head	AP	0.91 (0.05)	1.95 (0.88)	0.88 (0.09)	2.30 (1.04)	0.90 (0.05)	2.43 (1.23)
		ML	0.72 (0.17)	1.43 (1.06)	0.76 (0.19)	1.64 (1.29)	0.92 (0.08)	1.71 (1.08)
	Right Shank Right Thigh Sternum Head	AP	0.73 (0.15)	3.34 (1.27)	0.73 (0.11)	3.72 (1.60)	0.74 (0.12)	3.65 (1.51)
		ML	0.74 (0.16)	1.61 (1.17)	0.73 (0.18)	1.83 (1.33)	0.89 (0.10)	1.90 (1.16)
	Right Shank Back Sternum Head	AP	0.81 (0.14)	2.60 (1.46)	0.75 (0.14)	3.42 (1.87)	0.73 (0.14)	3.80 (1.94)
		ML	0.77 (0.17)	1.65 (1.90)	0.81 (0.18)	1.69 (1.93)	0.90 (0.16)	1.99 (1.58)
	Right Thigh Back Sternum	AP	0.88 (0.08)	3.32 (2.72)	0.83 (0.09)	3.57 (1.93)	0.86 (0.08)	3.87 (3.14)

	Sensor Placement	Direction	Shoulder-Width Stance Correlation (r) Mean (SD)	Shoulder-Width Stance RMSE (mm) Mean (SD)	Hip-Width Stance Correlation (r) Mean (SD)	Hip-Width Stance RMSE (mm) Mean (SD)	Narrow-Width Stance Correlation (r) Mean (SD)	Narrow-Width Stance RMSE (mm) Mean (SD)
	Head	ML	0.60 (0.26)	1.67 (0.88)	0.66 (0.28)	2.11 (1.52)	0.86 (0.12)	2.16 (1.32)
**5 IMUs**	Left Shank Right Shank Right Thigh Back Sternum	AP	0.92 (0.04)	1.80 (0.73)	0.91 (0.05)	1.91 (0.79)	0.92 (0.04)	2.05 (1.11)
		ML	0.86 (0.14)	0.89 (0.82)	0.90 (0.07)	0.92 (0.53)	0.97 (0.02)	1.08 (0.75)
	Left Shank Right Shank Right Thigh Back Head	AP	0.91 (0.04)	1.90 (0.80)	0.89 (0.07)	2.07 (1.49)	0.92 (0.05)	2.14 (1.19)
		ML	0.84 (0.15)	0.90 (0.82)	0.89 (0.09)	0.95 (0.64)	0.97 (0.02)	1.11 (0.75)
	Left Shank Right Shank Right Thigh Sternum Head	AP	0.74 (0.14)	3.24 (1.14)	0.74 (0.11)	3.61 (1.15)	0.78 (0.12)	3.30 (1.32)
		ML	0.81 (0.13)	1.25 (0.80)	0.84 (0.10)	1.25 (0.65)	0.95 (0.03)	1.36 (0.74)
	Left Shank Right Shank Back Sternum Head	AP	0.86 (0.10)	2.18 (1.15)	0.82 (0.10)	2.80 (1.53)	0.83 (0.12)	2.87 (1.65)
		ML	0.88 (0.12)	0.91 (1.01)	0.92 (0.05)	0.88 (0.70)	0.97 (0.03)	1.12 (0.78)
	Left Shank Right Thigh Back Sternum Head	AP	0.90 (0.05)	2.05 (0.91)	0.89 (0.05)	2.13 (1.60)	0.90 (0.06)	2.38 (1.33)
		ML	0.74 (0.22)	1.17 (0.91)	0.81 (0.14)	1.30 (0.70)	0.94 (0.04)	1.38 (0.99)
	Right Shank Right Thigh Back Sternum Head	AP	0.91 (0.04)	1.85 (0.82)	0.89 (0.07)	2.14 (1.78)	0.89 (0.04)	2.38 (1.18)
		ML	0.74 (0.16)	1.41 (1.08)	0.77 (0.19)	1.62 (0.71)	0.92 (0.08)	1.69 (1.09)
**6 IMUs**	Left Shank Right Shank Right Thigh Back Sternum Head	AP	0.92 (0.03)	1.76 (0.74)	0.91 (0.05)	1.90 (0.90)	0.92 (0.04)	2.02 (1.11)
		ML	0.85 (0.14)	0.89 (0.82)	0.90 (0.08)	0.92 (0.73)	0.97 (0.02)	1.08 (0.75)

Table S2. Center of mass estimation summary measures: no rigid body. Summary measures for each of the inertial measurement unit set-up conditions across the different stance widths, without the large low back rigid body. IMU: Inertial measurement unit; AP: anteroposterior; ML: mediolateral; r: Pearson's Linear Correlation Coefficient; RMSE: root mean square error; mm: millimetres; SD: standard deviation.

	Sensor Placement	Direction	Shoulder-Width Stance		Hip-Width Stance		Narrow-Width Stance	
			Correlation (r) Mean (SD)	***RMSE (mm)*** Mean (SD)	***Correlation (r)*** Mean (SD)	***RMSE (mm)*** Mean (SD)	***Correlation (r)*** Mean (SD)	***RMSE (mm)*** Mean (SD)
**1 IMU**	Shank	AP	0.72 (0.20)	3.59 (2.21)	0.69 (0.17)	4.39 (3.22)	0.53 (0.24)	5.39 (2.91)
		ML	0.74 (0.28)	2.50 (2.25)	0.77 (0.21)	2.43 (2.27)	0.89 (0.12)	2.38 (1.60)
	Right Thigh	AP	0.93 (0.04)	4.44 (4.26)	0.87 (0.15)	5.70 (8.68)	0.87 (0.06)	3.58 (1.94)
		ML	0.62 (0.21)	2.47 (1.67)	0.66 (0.29)	2.91 (2.76)	0.80 (0.18)	2.78 (1.85)
	Back	AP	0.55 (0.25)	5.39 (2.04)	0.50 (0.29)	6.92 (6.13)	0.60 (0.23)	5.87 (2.38)
		ML	0.17 (0.28)	2.74 (1.17)	0.47 (0.18)	2.51 (1.36)	0.78 (0.14)	2.66 (1.37)
	Sternum	AP	0.22 (0.36)	13.43 (5.90)	0.08 (0.34)	16.98 (9.29)	0.28 (0.26)	13.15 (5.61)
		ML	0.17 (0.29)	5.10 (1.46)	0.31 (0.12)	5.72 (2.43)	0.46 (0.17)	5.54 (2.10)
	Head	AP	0.16 (0.26)	18.91 (9.72)	0.10 (0.26)	21.90 (15.35)	0.21 (0.24)	21.69 (12.29)
		ML	-0.03 (0.32)	9.75 (3.81)	0.23 (0.19)	8.38 (3.68)	0.25 (0.18)	8.18 (3.13)
**2 IMUs**	Left Shank Right Shank	AP	0.79 (0.20)	3.02 (2.12)	0.81 (0.14)	3.52 (2.92)	0.71 (0.16)	3.94 (2.11)
		ML	0.83 (0.19)	1.36 (0.85)	0.90 (0.07)	1.22 (0.85)	0.96 (0.04)	1.33 (0.72)
	Right Shank Right Thigh	AP	0.94 (0.03)	2.62 (2.11)	0.89 (0.13)	3.36 (4.92)	0.89 (0.05)	2.22 (1.05)
		ML	0.72 (0.21)	2.08 (1.36)	0.73 (0.23)	2.32 (1.93)	0.88 (0.10)	2.19 (1.37)
	Left Shank Right Thigh	AP	0.93 (0.04)	2.69 (2.04)	0.91 (0.11)	3.42 (4.70)	0.90 (0.07)	2.33 (1.25)
		ML	0.76 (0.14)	1.53 (0.96)	0.81 (0.16)	1.63 (1.13)	0.92 (0.07)	1.59 (0.92)
	Right Shank Back	AP	0.83 (0.16)	2.62 (1.70)	0.79 (0.15)	3.06 (2.07)	0.68 (0.17)	3.94 (2.13)
		ML	0.76 (0.29)	1.85 (1.80)	0.79 (0.20)	1.85 (1.85)	0.92 (0.09)	1.81 (1.29)
	Right Shank Sternum	AP	0.62 (0.25)	4.12 (2.33)	0.54 (0.19)	5.48 (3.80)	0.55 (0.21)	4.95 (2.28)
		ML	0.75 (0.24)	2.05 (1.85)	0.76 (0.18)	2.25 (1.77)	0.90 (0.10)	2.00 (1.32)
	Right Shank Head	AP	0.71 (0.22)	3.61 (2.24)	0.70 (0.18)	4.21 (3.01)	0.55 (0.22)	5.22 (2.77)
		ML	0.74 (0.27)	2.32 (2.12)	0.78 (0.20)	2.30 (2.15)	0.90 (0.12)	2.25 (1.58)
	Right Thigh Back	AP	0.93 (0.03)	3.65 (3.24)	0.85 (0.20)	4.86 (6.76)	0.88 (0.06)	3.05 (1.68)
		ML	0.64 (0.22)	2.02 (1.43)	0.69 (0.28)	2.40 (2.21)	0.83 (0.16)	2.29 (1.53)
	Right Thigh Sternum	AP	0.80 (0.15)	4.75 (3.26)	0.72 (0.21)	5.76 (7.32)	0.77 (0.14)	3.98 (1.86)
		ML	0.62 (0.19)	2.22 (1.30)	0.65 (0.24)	2.70 (2.27)	0.80 (0.17)	2.50 (1.52)
	Right Thigh Head	AP	0.93 (0.04)	4.30 (4.03)	0.86 (0.16)	5.54 (8.27)	0.87 (0.08)	3.54 (2.00)
		ML	0.61 (0.22)	2.35 (1.59)	0.67 (0.29)	2.81 (2.65)	0.80 (0.18)	2.68 (1.81)
	Back Sternum	AP	0.54 (0.25)	5.41 (1.90)	0.48 (0.29)	6.79 (5.49)	0.60 (0.23)	5.78 (2.24)
		ML	0.18 (0.29)	2.73 (1.15)	0.47 (0.17)	2.51 (1.33)	0.78 (0.14)	2.69 (1.38)
	Back Head	AP	0.55 (0.24)	5.35 (2.02)	0.50 (0.29)	6.88 (6.14)	0.60 (0.23)	5.80 (2.36)
		ML	0.17 (0.28)	2.73 (1.17)	0.48 (0.18)	2.50 (1.36)	0.78 (0.14)	2.66 (1.37)
	Sternum Head	AP	0.22 (0.36)	13.37 (5.86)	0.08 (0.34)	16.86 (9.18)	0.28 (0.26)	13.07 (5.59)
		ML	0.17 (0.30)	5.09 (1.46)	0.31 (0.12)	5.70 (2.41)	0.46 (0.17)	5.53 (2.09)

	Sensor Placement	Direction	Shoulder-Width Stance		Hip-Width Stance		Narrow-Width Stance	
			Correlation (r) Mean (SD)	***RMSE (mm)*** Mean (SD)	***Correlation (r)*** Mean (SD)	***RMSE (mm)*** Mean (SD)	***Correlation (r)*** Mean (SD)	***RMSE (mm)*** Mean (SD)
**3 IMUs**	Left Shank Right Shank Right Thigh	AP	0.95 (0.03)	2.43 (2.02)	0.91 (0.12)	3.15 (4.80)	0.93 (0.04)	1.85 (0.95)
		ML	0.83 (0.14)	1.24 (0.71)	0.88 (0.10)	1.22 (0.73)	0.95 (0.04)	1.28 (0.66)
	Left Shank Right Shank Back	AP	0.87 (0.13)	2.18 (1.54)	0.87 (0.14)	2.43 (1.75)	0.82 (0.14)	2.86 (1.57)
		ML	0.87 (0.20)	0.89 (0.73)	0.94 (0.05)	0.87 (0.81)	0.97 (0.03)	0.97 (0.66)
	Left Shank Right Shank Sternum	AP	0.64 (0.22)	3.79 (2.13)	0.61 (0.21)	5.01 (3.82)	0.66 (0.18)	4.02 (1.91)
		ML	0.81 (0.18)	1.24 (0.76)	0.87 (0.08)	1.40 (0.77)	0.95 (0.03)	1.32 (0.62)
	Left Shank Right Shank Head	AP	0.78 (0.20)	3.03 (2.05)	0.80 (0.16)	3.43 (2.75)	0.72 (0.17)	3.86 (2.10)
		ML	0.83 (0.19)	1.28 (0.77)	0.91 (0.07)	1.14 (0.79)	0.96 (0.04)	1.22 (0.71)
	Left Shank Right Thigh Back	AP	0.93 (0.05)	2.01 (1.22)	0.88 (0.14)	2.61 (2.84)	0.90 (0.07)	2.16 (1.07)
		ML	0.77 (0.17)	1.21 (0.89)	0.83 (0.16)	1.37 (1.02)	0.94 (0.06)	1.33 (0.83)
	Left Shank Right Thigh Sternum	AP	0.76 (0.17)	3.55 (1.67)	0.69 (0.21)	4.73 (4.05)	0.77 (0.13)	3.34 (1.38)
		ML	0.73 (0.16)	1.49 (0.77)	0.78 (0.14)	1.77 (1.04)	0.90 (0.07)	1.62 (0.80)
	Left Shank Right Thigh Head	AP	0.93 (0.04)	2.59 (1.79)	0.89 (0.11)	3.36 (4.32)	0.89 (0.07)	2.42 (1.18)
		ML	0.74 (0.16)	1.48 (0.93)	0.82 (0.15)	1.59 (1.08)	0.92 (0.07)	1.51 (0.92)
	Right Shank Right Thigh Back	AP	0.94 (0.04)	1.95 (1.30)	0.89 (0.14)	2.47 (2.98)	0.90 (0.04)	2.04 (0.96)
		ML	0.74 (0.25)	1.59 (1.25)	0.77 (0.21)	1.80 (1.54)	0.92 (0.08)	1.71 (1.10)
	Right Shank Right Thigh Sternum	AP	0.75 (0.18)	3.57 (2.04)	0.67 (0.22)	4.81 (4.00)	0.74 (0.15)	3.36 (1.34)
		ML	0.73 (0.20)	1.80 (1.28)	0.74 (0.19)	2.14 (1.58)	0.89 (0.09)	1.94 (1.12)
	Right Shank Right Thigh Head	AP	0.92 (0.06)	2.64 (1.94)	0.88 (0.13)	3.25 (4.51)	0.88 (0.05)	2.33 (0.95)
		ML	0.72 (0.20)	1.93 (1.29)	0.75 (0.22)	2.20 (1.84)	0.88 (0.10)	2.08 (1.35)
	Right Shank Back Sternum	AP	0.81 (0.16)	2.73 (1.75)	0.77 (0.15)	3.27 (2.09)	0.68 (0.17)	3.97 (2.10)
		ML	0.77 (0.29)	1.83 (1.82)	0.79 (0.20)	1.85 (1.84)	0.92 (0.09)	1.79 (1.30)
	Right Shank Back Head	AP	0.82 (0.17)	2.63 (1.73)	0.79 (0.15)	3.05 (2.07)	0.68 (0.17)	3.93 (2.11)
		ML	0.76 (0.29)	1.83 (1.80)	0.79 (0.20)	1.84 (1.84)	0.92 (0.09)	1.80 (1.30)
	Right Shank Sternum Head	AP	0.62 (0.24)	4.08 (2.33)	0.55 (0.18)	5.38 (3.69)	0.56 (0.20)	4.91 (2.27)
		ML	0.75 (0.24)	2.03 (1.84)	0.76 (0.18)	2.23 (1.76)	0.90 (0.10)	1.99 (1.33)
	Right Thigh Back Sternum	AP	0.92 (0.04)	3.67 (3.20)	0.85 (0.18)	4.70 (6.77)	0.88 (0.07)	3.02 (1.61)
		ML	0.65 (0.21)	2.01 (1.42)	0.69 (0.28)	2.40 (2.23)	0.83 (0.16)	2.29 (1.53)
	Right Thigh Back Head	AP	0.93 (0.03)	3.61 (3.24)	0.85 (0.20)	4.83 (6.75)	0.89 (0.06)	3.02 (1.68)
		ML	0.64 (0.22)	2.01 (1.42)	0.69 (0.28)	2.40 (2.21)	0.83 (0.16)	2.29 (1.54)
	Right Thigh Sternum Head	AP	0.80 (0.13)	4.68 (3.25)	0.72 (0.20)	5.66 (7.27)	0.77 (0.14)	3.92 (1.88)
		ML	0.62 (0.19)	2.21 (1.30)	0.66 (0.25)	2.69 (2.26)	0.80 (0.17)	2.50 (1.52)
	Back Sternum Head	AP	0.54 (0.25)	5.37 (1.92)	0.49 (0.28)	6.77 (5.64)	0.60 (0.23)	5.73 (2.25)
		ML	0.18 (0.28)	2.72 (1.15)	0.48 (0.17)	2.50 (1.33)	0.78 (0.14)	2.68 (1.37)

	Sensor Placement	Direction	Shoulder-Width Stance		Hip-Width Stance		Narrow-Width Stance	
			Correlation (r) Mean (SD)	***RMSE (mm)*** Mean (SD)	***Correlation (r)*** Mean (SD)	***RMSE (mm)*** Mean (SD)	***Correlation (r)*** Mean (SD)	***RMSE (mm)*** Mean (SD)
**4 IMUs**	Left Shank Right Shank Right Thigh Back	AP	0.95 (0.02)	1.66 (1.12)	0.91 (0.14)	2.20 (2.90)	0.93 (0.05)	1.63 (0.76)
		ML	0.86 (0.17)	0.86 (0.64)	0.91 (0.08)	0.93 (0.70)	0.97 (0.03)	0.98 (0.63)
	Left Shank Right Shank Right Thigh Sternum	AP	0.77 (0.17)	3.38 (1.83)	0.70 (0.22)	4.61 (4.01)	0.78 (0.14)	3.03 (1.32)
		ML	0.80 (0.15)	1.20 (0.62)	0.84 (0.10)	1.43 (0.71)	0.94 (0.03)	1.33 (0.58)
	Left Shank Right Shank Right Thigh Head	AP	0.94 (0.03)	2.39 (1.78)	0.90 (0.12)	3.07 (4.39)	0.92 (0.04)	1.95 (0.87)
		ML	0.82 (0.15)	1.17 (0.66)	0.89 (0.09)	1.16 (0.68)	0.95 (0.04)	1.19 (0.66)
	Left Shank Right Shank Back Sternum	AP	0.86 (0.13)	2.28 (1.59)	0.85 (0.13)	2.65 (1.84)	0.81 (0.14)	2.88 (1.52)
		ML	0.87 (0.20)	0.86 (0.73)	0.94 (0.05)	0.86 (0.78)	0.98 (0.02)	0.95 (0.65)
	Left Shank Right Shank Back Head	AP	0.87 (0.14)	2.18 (1.56)	0.87 (0.14)	2.42 (1.77)	0.82 (0.14)	2.84 (1.55)
		ML	0.87 (0.20)	0.87 (0.73)	0.94 (0.05)	0.86 (0.80)	0.98 (0.03)	0.95 (0.66)
	Left Shank Right Shank Sternum Head	AP	0.65 (0.22)	3.74 (2.12)	0.62 (0.20)	4.90 (3.71)	0.66 (0.17)	3.97 (1.90)
		ML	0.81 (0.18)	1.23 (0.75)	0.87 (0.08)	1.38 (0.76)	0.95 (0.03)	1.30 (0.62)
	Left Shank Right Thigh Back Sternum	AP	0.92 (0.06)	2.07 (1.22)	0.88 (0.13)	2.57 (2.91)	0.89 (0.07)	2.13 (0.98)
		ML	0.77 (0.16)	1.19 (0.86)	0.84 (0.15)	1.37 (1.01)	0.93 (0.06)	1.33 (0.82)
	Left Shank Right Thigh Back Head	AP	0.93 (0.05)	1.97 (1.22)	0.88 (0.14)	2.60 (2.83)	0.90 (0.08)	2.12 (1.08)
		ML	0.77 (0.17)	1.20 (0.88)	0.83 (0.15)	1.36 (1.01)	0.94 (0.06)	1.32 (0.83)
	Left Shank Right Thigh Sternum Head	AP	0.77 (0.16)	3.48 (1.64)	0.70 (0.21)	4.62 (3.96)	0.77 (0.13)	3.27 (1.38)
		ML	0.73 (0.16)	1.48 (0.78)	0.78 (0.14)	1.76 (1.03)	0.90 (0.07)	1.61 (0.80)
	Right Shank Right Thigh Back Sternum	AP	0.93 (0.05)	2.03 (1.37)	0.89 (0.14)	2.49 (3.01)	0.90 (0.04)	2.03 (0.87)
		ML	0.76 (0.24)	1.57 (1.27)	0.78 (0.22)	1.78 (1.56)	0.92 (0.08)	1.70 (1.10)
	Right Shank Right Thigh Back Head	AP	0.94 (0.05)	1.94 (1.32)	0.89 (0.14)	2.45 (2.96)	0.90 (0.04)	2.01 (0.94)
		ML	0.75 (0.25)	1.57 (1.24)	0.77 (0.21)	1.79 (1.53)	0.92 (0.08)	1.70 (1.11)
	Right Shank Right Thigh Sternum Head	AP	0.76 (0.18)	3.52 (2.03)	0.68 (0.21)	4.70 (3.91)	0.74 (0.14)	3.31 (1.31)
		ML	0.74 (0.20)	1.78 (1.27)	0.74 (0.19)	2.13 (1.57)	0.89 (0.09)	1.93 (1.12)
	Right Shank Back Sternum Head	AP	0.81 (0.17)	2.71 (1.78)	0.78 (0.15)	3.21 (2.07)	0.68 (0.17)	3.95 (2.09)
		ML	0.77 (0.28)	1.81 (1.81)	0.79 (0.20)	1.84 (1.84)	0.92 (0.09)	1.79 (1.31)
	Right Thigh Back Sternum Head	AP	0.92 (0.04)	3.62 (3.22)	0.85 (0.19)	4.69 (6.75)	0.88 (0.06)	3.00 (1.63)
		ML	0.64 (0.21)	2.00 (1.41)	0.69 (0.28)	2.39 (2.22)	0.83 (0.16)	2.29 (1.53)

	Sensor Placement	Direction	Shoulder-Width Stance		Hip-Width Stance		Narrow-Width Stance	
			Correlation (r) Mean (SD)	***RMSE (mm)*** Mean (SD)	***Correlation (r)*** Mean (SD)	***RMSE (mm)*** Mean (SD)	***Correlation (r)*** Mean (SD)	***RMSE (mm)*** Mean (SD)
**5 IMUs**	Left Shank<br>Right Shank<br>Right Thigh<br>Back<br>Sternum	AP	0.94 (0.04)	1.75 (1.18)	0.91 (0.13)	2.19 (2.90)	0.93 (0.05)	1.59 (0.68)
		ML	0.87 (0.16)	0.83 (0.62)	0.92 (0.08)	0.93 (0.70)	0.97 (0.03)	0.97 (0.61)
	Left Shank<br>Right Shank<br>Right Thigh<br>Back<br>Head	AP	0.96 (0.02)	1.64 (1.12)	0.91 (0.14)	2.18 (4.01)	0.94 (0.05)	1.58 (0.76)
		ML	0.86 (0.16)	0.84 (0.63)	0.92 (0.08)	0.93 (0.71)	0.97 (0.03)	0.97 (0.63)
	Left Shank<br>Right Shank<br>Right Thigh<br>Sternum<br>Head	AP	0.77 (0.16)	3.32 (1.81)	0.71 (0.21)	4.50 (4.39)	0.79 (0.13)	2.97 (1.29)
		ML	0.80 (0.15)	1.19 (0.62)	0.85 (0.09)	1.41 (0.68)	0.94 (0.03)	1.32 (0.58)
	Left Shank<br>Right Shank<br>Back<br>Sternum<br>Head	AP	0.86 (0.14)	2.26 (1.60)	0.86 (0.13)	2.58 (1.84)	0.81 (0.14)	2.86 (1.52)
		ML	0.87 (0.20)	0.85 (0.73)	0.94 (0.05)	0.86 (0.78)	0.98 (0.02)	0.94 (0.65)
	Left Shank<br>Right Thigh<br>Back<br>Sternum<br>Head	AP	0.93 (0.05)	2.02 (1.22)	0.88 (0.13)	2.56 (1.77)	0.90 (0.08)	2.10 (1.02)
		ML	0.77 (0.16)	1.19 (0.86)	0.84 (0.14)	1.36 (0.80)	0.93 (0.06)	1.32 (0.83)
	Right Shank<br>Right Thigh<br>Back<br>Sternum<br>Head	AP	0.93 (0.05)	2.01 (1.37)	0.89 (0.14)	2.46 (3.71)	0.90 (0.04)	2.01 (0.88)
		ML	0.76 (0.24)	1.55 (1.26)	0.78 (0.21)	1.78 (0.76)	0.92 (0.08)	1.70 (1.11)
**6 IMUs**	Left Shank<br>Right Shank<br>Right Thigh<br>Back<br>Sternum<br>Head	AP	0.95 (0.03)	1.71 (1.17)	0.91 (0.13)	2.17 (2.91)	0.94 (0.05)	1.55 (0.71)
		ML	0.86 (0.16)	0.83 (0.62)	0.92 (0.08)	0.92 (1.01)	0.97 (0.03)	0.96 (0.61)

Table S3. Center of mass estimation summary measures: rigid body. Summary measures for each of the inertial measurement unit set-up conditions across the different stance widths, with the large low back rigid body. IMU: Inertial measurement unit; AP: anteroposterior; ML: mediolateral; r: Pearson's Linear Correlation Coefficient; RMSE: root mean square error; mm: millimetres; SD: standard deviation.

References

1. Gui P, Tang L, Mukhopadhyay S. MEMS based IMU for tilting measurement: Comparison of complementary and Kalman filter based data fusion. 2015 IEEE 10th Conference on Industrial Electronics and Applications (ICIEA). 2015. pp. 2004–2009. doi:10.1109/ICIEA.2015.7334442

2. Legani G, Adamini R, Zappa B. SpaceLib. Brescia, Italy; 2005.

3. Zatsiorsky VM, King DL. An algorithm for determining gravity line location from posturographic recordings. Journal of Biomechanics. 1997;31: 161–164. doi:10.1016/S0021-9290(97)00116-4

4. Caron O, Faure B, Brenière Y. Estimating the centre of gravity of the body on the basis of the centre of pressure in standing posture. Journal of Biomechanics. 1997;30: 1169–1171. doi:10.1016/S0021-9290(97)00094-8

5. Mengarelli A, Cardarelli S, Tigrini A, Marchesini L, Strazza A, Fioretti S, et al. Inertial sensor based estimation of the center of pressure during unperturbed upright stance. 2019 IEEE 23rd International Symposium on Consumer Technologies, ISCT 2019. Institute of Electrical and Electronics Engineers Inc.; 2019. pp. 186–191. doi:10.1109/ISCE.2019.8900980

6. Najafi B, Lee-Eng J, Wrobel JS, Goebel R. Estimation of center of mass trajectory using wearable sensors during golf swing. Journal of Sports Science and Medicine. 2015;14: 354–363.
